# Medical Items and News of Interest to the Physician

**Published:** 1875-07

**Authors:** 


### For the Use of Physicians.

CULLED FROM THE STANDARD MEDICAL JOURNALS OF THE WORLD.

NOTE.—These formulæ are intended only for the regular practitioner of medicine, and should be employed only by him, or under his immediate supervision.

**Local Anæsthesia**

May, it is said, be obtained by rubbing for a minute the part upon which it is desired to operate, with a mixture of powdered camphor, two and a half drachms, and sulphuric ether, five drachms.

**Sulphate of Cadmium in Gonorrhea.**

Dr. Gazean has employed, with much success, in the acute stage of urethral blennorrheas, a solution of the sulphate of cadmium, one grain to two, three or four ounces of water, used every two hours.

**Wine of Beef and Iron.**

Wine of orange, 1 pint.
Extract of beef, 4 drachms.
Ammonio-citrate of iron, 128 grains.

Mix. Some use sweetened sherry wine instead of orange wine.

**Iodine Caustic.**

Iodine caustic is prepared by dissolving four grammes of iodine in eight grammes of glycerine. It is used in lupus by applying it once every other day, and covering the parts with gutta-percha.—This treatment is continued for several weeks.—*Canada Lancet.*

**Ozœna.**

At the Detroit Medical Society, Dr. Lathrop related a case of ozœna, in which the patient found complete relief in the persistent use of new milk and common salt—about a teaspoonful of salt disolved in a pint of milk. The remedy is one that commends itself to notice by its simplicity.

**Anti-Constipation Pills.**

℞

Juglandin, gr. xxv.
Strychnia, gr. ij
Leptandrin, gr. xxv.
Ext. hyoscyamus, gr. xij.

With castile soap and ol. menthæ pip, a mass is made and divided into one hundred pills, of which two or three are a dose.

**"Dead Shot Pill, for the Same."**

Take sulphate of quinine, ℨ j;
Reduced iron, ℨ jss;
Strychnia;
Arsenious acid, āā. gr. iij;
Confection of rose, sufficient.
Make into sixty pills.

*Druggists' Circular.*

**Snow as a Hæmostatic.**

Dr. Hane (*Centrablatt fur Chirurgie*, No. 38, 1874) recommends very highly snow as a means of preventing hemorrhage in those operations in which Esmarch's method cannot be used. A handful of dry snow pressed upon the bleeding wound he found in a case of tracheotomy to act most favorably, soaking up the blood like a sponge and arresting the bleeding.

**Hemorrhoids Treated with Ergot.**

Dr. G. W. Semple recommends the injection of ergot into the rectum for the treatment of hemorrhoids. A half fluid drachm of the extract was used in half an ounce of water. He refers to Langenbeck's method of injecting ergotin underneath the mucous coat of the rectum, but claims that his method has the advantage of painlessness and facility of employment by the patient himself.—*Virginia Med. Record.*

**To Prevent Surgical Fever.**

The following is the mixture used by Prof. Pancoast in surgical cases where he fears surgical fever:

℞

Aquæ camphoræ, f. ℥ ij.
Spir. mindereri, f. ℥ jss.
Spir. eth. nit., f. ℥ iij.
Antimonii tartarisatæ, gr. ss.
Morphiæ acetat, gr. ss, or more as needed.

Mix.

Sig. A teaspoonful every hour while awake.

**Nelaton's Method Resuscitation from Chloroform Narcosis.**

Dr. J. Marion Sims most impressively details his observations respecting the practical workings of the above method. Briefly stated, it consists of in reversing the body of the patient, either by hanging him up by the feet or laying him over a bed or table, so that the blood from the greater part of the body can gravitate towards the head. The tongue is drawn forward by a tenaculum and artificial respiration tried. This practice is based on the theory that death from chloroform takes place from cerebral anæmia.

**A Local Anodyne.**

The *London Lancet* gives the following formula for a convenient local anodyne: Take of elastic collodion one ounce; hydrochlorate of morphia, fifteen grains. Dissolve the morphine salt in the collodion. Spread with a camel's-hair brush some of this solution on the painful part, and place some oiled silk over the spot. The effect is stated to be most satisfactory.

**Hypodermic Injection of Ergot.**

Dr. P. C. Williams reports three cases of post-partum hemorrhage treated successfully by means of the hypodermic injection of ergot. He advocates this method as prompt, safe and efficient.—No abscesses or other bad effects resulted. He used the fluid extract, but also suggests using Squibb's solid extract for this purpose, the extract having been rubbed up with water in the proportion of a grain to the minim.—*St. Louis Med. & Surg. Jour.*

**The Sulphides in Boils.**

Mr. Louis Lewis, writing to *The Lancet*, says: A patient applied to me, suffering from an eruption of boils, which appeared in successive crops, and exuded a thin ichorous pus. I prescribed the sulphide of calcium in small frequently repeated doses, (not more than a grain altogether during each day). It was given simply in water, and no other medicine was used. In a few days the pus became creamy and laudable, the inflammation became limited in area, and the boils rapidly died away, no fresh crop appearing.

**Cold Powder.**

We have long been in the habit of using what we call a *cold powder*, which we have found of great value in breaking up colds when taken in time, and in modifying their force when taken late.

The prescription is as follows:

Camphor, five parts. Dissolve in ether to the consistence of cream. Then add carbonate of ammonia, four parts; opium-powder, one part.

Mix and keep in a tightly-corked bottle. The dose is of course regulated by the opium, and ranges between three and ten or fifteen grains. We have been accustomed to prescribe it for our friends by the finger-nail full, or as much as one can put on the finger-nail.

This powder may be taken in a little water just before retiring, by preference, or at any hour of the day, or whenever there is a suspicion of having caught cold. If need be a moderate dose may be taken several days in succession.

**Liquid Dentifrice.**

The same journal gives the following as a valuable liquid dentifrice.

Fine potash soap, ℥ iij;
Cream of tartar, ʒ j.
Alcohol, sp. gr. 0.910, ℥ xviij.
Distilled water of any desired perfume, ℥ vj.

Digest and filter.

**Lotion for Prurigo.**

Lemon-juice 10, aromatic vinegar 5, and water 200 parts. To be used several times a day in order to relieve the irritation of pruritus of the vulva or scrotum. After each application the skin to be dried and covered with potato starch or lycopodium. Frequent baths should be taken, and alcoholic drinks to be abstained from.—*Union Medicale.*

**For Rheumatism.**

Said to have been prescribed with specific effect in many cases:

Take Iodide of potassium, ʒ ijss;
Tincture of cimcifuga, f. ℥ jss;
Wine of colchicum seed, f. ℥ j;
Fl. ext. henbane, f. ℥ ss;
Simple syrup, f. ℥j.

Dose; A teaspoonful, copiously diluted with water, every four hours.

**Croton Oil Paint.**

Dr. John W. Corson recommends, as a sedative for blisters, the application of the following, called mild croton oil paint:

℞
Croton oil, 1 part.
Strong ether, 2 parts.
Tinct. iodine, 5 parts. M.

Sig. Apply two or three coats at a time, with a camel's-hair brush, over a small surface, once a week. This is useful for children, females and sensitive males.

**A Cheap Fumigator.**

The following will be found to be a cheap and pleasant fumigator for sick-rooms, diffusing a heathful, agreeable, and highly penetrating disinfectant odor in close apartments, or wherever the air is deteriorated. Pour common vinegar on powdered chalk until effervescence ceases, leave the whole to settle, and pour off the liquid. Dry the sediment and place it in a shallow earthen or glass dish, and pour upon it sulphuric acid until white fumes commence arising. This vapor quickly spreads, is very agreeably pungent, and acts as a powerful purifier of vitiated air.

**Carbolic Acid Inhalations.**

Dr. Rothe commends carbolic acid inhalations in phthisis, and gives the following formula:

℞
Carbolic acid in crystals.
Spirits of wine āā 2 parts.
Tincture of iodine, 1 part.
Distilled water, 10 parts. M.

Twenty-five to thirty drops to be added to one or two tablespoonfuls of water, for inhalation. He uses the same solution for diptheria to paint the tonsils, pharynx, etc.

---

**Cauterization in Hydrocele.**

Dr. Vallette, of Lyons, recommends cauterization for the radical cure of hydrocele, which he has practised forty times, with success almost constant. He perforates the integuments with one of the branches of the caustic holder, which has a sharp point, and thrusts it into the venous bundle, whilst the other branch is placed behind the veins—the two grooves having first of all been filled with caustic, all brought together by means of a screw. This instrument remains *in situ* for forty-eight hours, and in a fortnight the cure is completed. In vaginal hæmatocele he traverses the pouch filled with blood with a seton dipped in caustic, and withdraws it next day; there arises after this a moderate, progressive inflammation, which gradually determines the resorption of the effused blood and modifies the walls of the sac.

---

**Anæsthesia by Injection of Chloral into the Veins.**

Professors Deneffe and Van Wetter report seven new cases in which operations have been successfully performed under the anæsthesia produced by the injection of chloral. They state that this plan has been successfully tried in eighteen cases, and they give the details of the last seven, one of which was by Ore, of Bordeaux, the originator of the plan.

Ore's case was a young man, twenty years of age, from whom a tuberculous testicle was to be removed. The steps of the operation were substantially as follows: It was commenced at 8:43 A. M., at which time the median basilic vein was punctured, and one minute later 30 grains of chloral had been injected; after two minutes the patient appeared sleepy; the pulse was 112. At 8:51 A. M., there was complete insensibility, and the operation was commenced, lasting seventeen minutes. At 8:59, A. M., there appeared to be some difficulty in breathing, from the relaxation of the tongue, which blocked up the posterior portion of the buccal cavity. By raising the hyoid bone and passing a current over the left hypogastric, this condition was overcome. The amount of chloral injected was about 100 grains, and the injection lasted seven minutes, producing complete anæsthesia, which lasted about an hour.—*Med. Record.*

Rather a dangerous proceeding, we should judge —cannot recommend it.—ED BISTOURY.

---

**Sciatica.**

Dr. I. Pretz (*Wiener Medizinische Presse*) gives as his personal experience the following facts: Having an attack of sciatica for six months, he tried all the ordinary remedies, with no avail. Observing that his attacks were slighter after eating, he determined to eat as often as the pain recurred. This was often twelve times in twenty-four hours. He constantly improved under this treatment, and in about two months entirely recovered. Two other cases were afterwards treated with similar results. He accounts for the result by the development of heat from the food and drink.

---

**An Improved Poultice.**

At a recent meeting of the Academie de Medcine, Paris, M. Le Fort read his report on a substitute for the ordinary linseed-meal poultice invented by M. Lelievre. It is prepared by saturating two superimposed layers of wadding with a solution of *Fucus Crispus*, or Carrageen lichen, and drying them in a stove after they had been submitted to strong pressure. In this way a sheet of the consistence of cardboard is produced, a portion of which is cut off when wanted, and soaked in hot water for fifteen or twenty minutes; This swells it out and fills its tissue with a mucilaginous fluid. It has been tried in several of the hospitals, to the great satisfaction of both patients and attendants. It can be prepared in large quantities beforehand, and will keep for a long time without undergoing any alteration. MM. Demarquay, Gosselin, and Verneuil pronounce it to be far superior to the linseed poultice; it keeps moist for more than sixteen or eighteen hours; it does not slip, is inodorous, does not readily ferment, nor does it soil the linen or bed of the patient. The new poultice is destined to render great service in hospitals and ambulances, and, above all, on board ship, where it is difficult to keep the linseed in a good state of preservation.

The foregoing appears in the *Homœpathic World.* The poultice produced as described seems very similar to the *Cataplasme Hamilton* which was introduced into French pharmacy a year or two ago.

**Calamus in Dysentery.**

In the dysentery of children Dr. Evers advocates the use of the following convenient mixture:

℞
Bruised calamus root, ℥ ij.
Coriander seed, ʒ j.
Black pepper, ℥ ss.
Water, Oj.

Boil to twelve ounces and cool. Dose for an adult, one ounce three times daily; for a child, one to three drachms, sweetened if preferred.

---

**The Cyanides in Rheumatism.**

In acute articular rheumatism, Dr. Luton has used two cyanic preparations, cyanide of potassium and cyanide of zinc. The latter is a white powder, inert, insoluble in water, tasteless, odorless and yet really powerful. It can be given in pills or, still better, suspended in mucilage. The doses are ¾ gr., 1½ gr., 2 gr., or even 3 gr., (5, 10, 15, 20 centigrammes) throughout the day. The cyanide of potassium is freely soluble, more active when properly prepared, and should be preferred. The dose is ¾ gr. to 1½ gr. (5 to 10 centigrammes) in a day. Dr. Luton has never exceeded 2 grs. (15 centigrammes,) which quanity he has observed to produce colic.

---

**Superior Cod-Liver Oil Mixture.**

Dr. Andrews, of the State Lunatic Asylum at Utica, has given a formula for preparing cod-liver oil which disguises its terrible taste, and places a valuable agent at our command in almost every case.

The chief objection to it is the fact that it is difficult to get the druggist to prepare it in the proper manner, because it requires so much time. When thoroughly prepared it will keep well for a long while.

FORMULA.

Yolk of eggs, No. iv.
Glycerine, ℥ i.
Cod-liver oil, ℥ iv.
Phosphoric acid, dilute, ℥ ss
Sherry wine, ℥ ij.
Essence of almonds, ʒ i.

The eggs and glycerine are first to be carefully triturated in a mortar, and while the trituration is continued add the oil, drop by drop. When this is done, the acid, wine, and essence may be added. The above is not the precise formula used at the Asylum, but it is essentially the same. The manner of making the emulsion is the important feature. The amount of phosphorus can be varied to suit diffierent cases, or other remedies may be added, such as arsenic, etc.—*Medical Record.*

**Hemorrhage in Extracting Teeth.**

For this difficulty Mr. Salter recommends the following:

℞

Vitelli ov., ij.

Olei terebinth, ʒ iss.

Sacchari, ʒ ij.

Tinct. ferri sesqui chloridi, ʒ iij.

Aquae, ℥ viij.

A tablespoonful to be taken every hour; or

℞

Tannin, gr. v.

Spt. vini rect., ʒ ss.

Aquae, ℥ iss.

To be taken every hour. As tannin is apt to produce nausea when taken on an empty stomach, he recommends its association with some (non-albuminous) food. In the irritability of sanguineous exhaustion, opiates may become necessary doses.

---

**On the Treatment of Epilepsy, both Nocturnal and Diurnal, with the Bromide Treatment.**

I notice a communication in the *Medical Record* of March 6, 1875, from Dr. James Gilliam La Roe, Jr., on his experience in treating a case of nocturnal epilepsy with bromide of potassium. I was for three years connected with the hospital for the insane at Mount Pleasant, Iowa, where we had quite a number of epileptics under treatment.

We never made any distinction in the treatment of the two forms of the disorder. We first turned our attention to the digestive apparatus, getting it into perfect order; keeping the patient upon light diet, and excluding all meats. We then commenced with Dr. Brown-Sequard's bromide ammonium mixture (the composition of which is known to the profession), beginning with ʒ i. ter die, and gradually increasing until "brominism" was produced; the convulsions generally ceased at this point, and did not again return so long as the treatment was continued. One patient we gave as high as ʒ iv. of the mixture three times daily ere the convulsions ceased to recur.

When the eruption appeared (which usually follows the long continued use of bromide of potassium), we lessened the dose.

We had one patient suffering from nocturnal epilepsy, who was quite violently insane when admitted to the hospital, but became quiet and free from epileptic paroxysms under the bromide treatment.

In all cases of epilepsy I regard it of eminent importance that the digestion of the patient be carefully observed and taken care of.—JOHN H. KULP, M. D., *in the Medical Record.*

**Hypodermic Injection of Ergotine in Varicocele.**

In a case of varicocele which had existed for a long time, Dr. Bertarelli, of Rome, injected a solution of ergotine under the skin of the scrotum. The solution consisted of ergotine, 1 gramme; water, with a little alcohol, 2 grammes. The patient was ordered to maintain absolute repose and to make local application of cold compresses. The next day the varicosities had disappeared. The success was complete after another injection, which was attended by but slight local reaction.

Dr. Cittaglia had cured another case of varicocele by the same treatment. By the eighteenth day nearly all the varicosities had disappeared; and there was nothing but a slight induration of the corresponding testicle to be observed.—*Alm. di Terapic.*

---

**Antagonism between Cloral Hydrate and Calabar.**

Dr. John Hughes Bennett (*British Medical Journal*, October, 1874) presents the experiments from which the following conclusions are drawn. Hydrate of chloral modifies to a great extent the action of a fatal dose of extract of calabar bean. This antagonism is limited by two conditions:

1. By the doses administered. More than a minimum fatal dose of extract of calabar bean destroys life, notwithstanding the administration of chloral.

2. By the interval of time between the administration of the two substances. There is great probability of saving life in those instances in which both substances are given almost simultaneously. This probability is diminished if the chloral be given five or eight minutes after the extract of calabar bean. But even in those cases in which death occurs after the introduction of both substances, the effects of the calabar bean are much less marked.

---

**Ringworm treated by Oleate of Mercury.**

The advantages which oleate of mercury, says Dr. Ed. Cane, in the London *Lancet*, seem to possess over other remedies are:—

1. It is a certain remedy, if carefully applied.

2. It produces no staining or injury of the skin. In cases where the disease appears on the face, it is of great importance to avoid any disfigurement of staining.

3. It is painless in its application. This is not the case with the ordinary strong parasiticides, most of which produce vesication, etc.

4. It readily penetrates into the sebaceous glands, hair-follicles, and even into the hairs themselves, the mercury being in a state of solution in an oily medium, and is therefore much more likely to destroy the fungus than the spirituous or aqueous solutions of mercury, etc. This penetrating power of the oleate may be increased by adding a small quantity of either (one part to eight) to it.

In very sensitive skins the irritation sometimes produced by it may be avoided by using a weaker solution (five per cent.), and by applying it with a camel's-hair brush. In slight cases this method is all that is necessary, but where the fungus has invaded the hair it is advisable to rub in the oleate gently.

---

**Burns and Scalds.**

Dr. L. R. Davidson writes to the *Boston Jour. of Chem.*, as follows:

Having seen so many valuable formulæ in your excellent journal, I enclose one to you that has been used in Bellevue Hospital, New York, with a great deal of success, for burns and scalds:

White glue, ℥ viiss.
Water, Oj.

Dissolve the glue in the water by means of a water bath, and then add

Glycerine, ℥ij.
Carbolic acid, ʒ ij.

This mixture should be kept in a cool place.—It is to be used with a brush as an outward application.

---

**Chocolate in Chronic Intestinal Catarrh.**

A series of articles from the pen of Dr. Karner have lately appeared in the *Allegimeine Wiener Medicinische Zeitung*, in which he shows the value of this substance as an article of food. He refers especially to its use in chronic intestinal catarrh, and cites, among others, the following typical case of chronic catarrh of the intestines to illustrate its action in the simplest manner: Rosalia M., aged seventeen months, poorly developed and nourished, suffered from intense meteorism, numerous thin, fluid, feculent discharges, which alternated from time to time with normal stools.—There was considerable emaciation, and the child also had intertrigo, of which there were frequent relapses. The diarrhœas could be attributed only to poor nourishment. After strictly regulating the diet, small doses of Dover's powder and acetate of lead were first administered, and in three days were substituted by the chocolate, of which a cup full was given daily. A dessert-spoonful of the powder sufficed for a cup of chocolate. The mother was also instructed to allow the child as little fluids to drink as possible. The result was astonishing. The discharges decreased in number day by day, the weight of the child rapidly increased, and after a few weeks it had perfectly recovered; a remarkable change for the better was observed in its bodily development, and the intertrigo had not recurred.

---

**Catarrh.**

Dr. C. S. Bull, of New York, writes as follows: I have used the tincture of the sesquichloride of iron quite frequently in cases of recent or incipient nasal catarrh, what is ordinarily called a cold in the head, and almost invariably with a rapid beneficial effect. I give it as soon as the patient complains of the sense of fullness in the nose and head, in doses of from ♏ xx. to ʒ ss, and repeat every hour; usually I have not been obliged to give more than four or five doses, and sometimes not more than two. I have taken it myself and always with good results. But it must be given early, otherwise it fails in producing the desired effect. I have not noticed any bad effects from its use in such frequently repeated doses.

Dr. N. B. Sizer, of Brooklyn, has during some time past, made brief notes of coryza treated by tincture of the chloride of iron, and recorded his results. He has left unrecorded a number of ordinary cases of cold in the head, neither severe or annoying to the patient, in all of which, according to his recollection, relief was prompt and decided. His results are as follows:

Number of cases recorded, 28. Age of youngest, 3 months; of oldest, 68 years.

Results:—

| | |
|---|---|
| Cured in 24 hours | 10 |
| " 36 " | 12 |
| " 48 " | 3 |
| Much relieved but not cured | 1 |
| Symptoms alleviated but not cured | 2 |
| | 28 |

In those not cured the catarrhal inflammation was modified somewhat, but the disease ran its usual course. There was no personal idiosyncrasys as far as known, to interfere with the curative results in these last cases, and the cause of failure is unknown. The dose varied from three to thirty-five minims, with an equal amount of glycerine. The thirty minim dose has been given to an infant, with wonderfully quick effect, done tentatively, however, and not as a usual thing.

There would seem to be no doubt, as far as my observation goes, of the generally excellent effect of the tincture in these cases. The tincture should be largely diluted with water, and taken through a glass tube or straw so as to protect the teeth. Its effect is more pleasant if the stomach is not entirely empty. The tendency of the iron to constipate may be very much lessened or prevented by the addition of three or four drops of the tincture of belladonna to each dose.

---

**Guarana in Headache.**

Dr. A. P. Brown, of Jefferson, Texas, writes to the *Medical and Surgical Reporter*, Philadelphia, as follows: "Guarana, which has been exciting some attention this last year or two, has proved, in my hands, one of the very best medicines in most all cases of headache, and I will here cite a few typical cases in which I have tried this, to me, new and valuable medicine. Mrs. S., aged seventy years, says she has had a headache for the last fifteen years, caused by cinchonism during malarial fever. I gave her guarana in five grain pills five times daily. She left here about three weeks ago, to go, in the cars, to her friends in Tennessee, and she sends word she is recovered from that dreadful trouble. Mrs. S., daughter-in-law of the last, has suffered with sick headache for a long period. I gave her the guarana in five grain pills every two hours, and she sends me word of her perfect and rapid relief, and says the medicine causes 'a delightful exaltation.'"

Case No. 3 is that of an old hard drinker, who has left off drinking any alcoholic beverages, but continues to smoke tobacco, and wakes every morning with a headache; gave mass hydrarg., one scruple at bedtime, with guarana pills, six grains every three hours; head relieved after a few doses. Guarana will not permanantly cure any case of headache, but will certainly relieve most cases, and that promptly; and where there is no return in the patient to the producing cause, I believe the headache will not return after the use of this medicine. I have not been able to observe any effect in the system except that of exhilarating nerve sedative.

---

**Rattle Snake Bite.**

The following occurs in the *Medical Times*, from the pen of Assistant-Surgeon Wilson, U. S. A.

Mr. John R. Magruder (son of the late Dr. Hezekiah Magruder, Washington, D. C.,) now residing about twenty miles from this post, engaged in mining and smelting copper, informed me about three months since that one of his Mexican laborers had been bitten in the calf of the leg by a rattlesnake. Another Mexican went out, looked about for a few minutes, found a plant, chewed up some of its leaves, and applied the bolus to the wound, when the swelling (which had become considerable) disappeared in a short time, and the man recovered.

I requested Mr. Magruder to procure me a specimen of this same plant, which I forwarded to Assistant-Surgeon, J. J. Woodward, U. S. A., Surgeon-General's Office, Washington, and he sent it on to Professor Asa Gray, of Cambridge, who identified it as the "Acerates decumbens," or the "Anantherix decumbens of Nuttal," a plant belonging to the milk-weed family.

My *experience* of its virtues is *nil*, as I have not been fortunate enough to see a case of rattlesnake-bite for a long time; but Mr. Magruder is a gentleman whom I have known intimately for the past two and a half years, and his statement is in every way reliable. I present it through your columns to the profession for what it is worth, and because I know from repeated inquiries I have made that the Mexicans depend upon it as an unfailing remedy. I would be very glad to have it tried in such cases, and the results published in some medical journal, for the benefit of myself and other members of the profession."

—We have seen an article in *Forest and Stream*, in which a sportsman reports that he has seen the *gaul* of the rattlesnake used upon the wound made by the reptile, with the effect of removing the poison and curing the patient. We have met with persons who have declared this remedy to be effectual. ED. BISTOURY.

---

**Whooping-Cough.**

Dr. J. W. Keating writes upon this subject to the *Medical Times*, as follows:

Believing that those more fortunate members of the profession who are placed by circumstances in a position to note the action of remedies in the treatment of epidemic forms of disease, should make known the results of their investigations, I beg leave to add my few drops to the great river of experience.

In the early summer months of this year, while resident physician in the children's ward of the Philadelphia Hospital, I had occasion to see an epidemic of measles and whooping-cough, which diseases occurred at the same time, and ran their course together. Owing to this fact, and also that, as we all know, the children are none of the strongest, the mortality was rather large—forty per cent. I was much interested at this time in the controversy as to the possibility, by medical means, of cutting short an attack of whooping-cough, and I availed myself of the uncomplicated cases to test the remedies proposed.

From the first, I found quinine to be the most reliable.

The number of cases was large, and, as is usual in a hospital, the number of nurses small, so that I was obliged to abandon the idea of noting the frequency of the paroxysms in every case, and could only limit myself to the few who had their mothers constantly with them, and where the intellectual capacity of the latter enabled them to interest themselves in my experiments.

As an example, I shall narrate one case which was particularly interesting, as the disease was extremely severe, and was uncomplicated. This child was fifteen months old, had been sleeping with its mother, who was an assistant nurse, in the room with the other children, most of whom had both whooping-cough and measles, and took whooping-cough, the attack of measles being deferred till a later period.

For twenty-four hours the mother carefully noted, by pin-holes in a card, the number of paroxysms. I then ordered one-half grain of quinine every hour during the day, the same dose to be given every two hours during the night. At the end of twenty-four hours I again had the "coughing-spells" noted. They had diminished in frequency exactly *one-half.* This experiment was often repeated, with the same results, until the end of the week, at which time the paroxysms were very few, but had not diminished in severity.

As an example of the same result in an older child, I may memtion the case of a girl about fifteen years of age, who came to Philadelphia suffering from a severe attack of pertussis. The child was particularly annoyed by the severe nocturnal coughing spells, which nothing seemed to relieve. I placed her upon the quinine treatment, and the result was really wonderful; I may say that after the first day she coughed but little, and in less than two weeks the disease had entirely disappeared.

In order to avoid repetition, the conclusions which I arrived at are given, as follows:

1. That in most cases quinine, given in solution, will diminish the frequency of the paroxysms of whooping-cough, provided it be given in sufficiently large doses.

2. That quinine can be given to children in proportunately much larger doses than to adults, but in very young infants it is contra-indicated, as it always causes vomiting.

3. That carbonate of ammonium will in almost all cases relieve the severity of the paroxysms, and consequently should be given in conjunction with quinine when this indication for its use exists.

4. That the dose of quinine for a child of two years should be at least ten grains daily, in divided doses; it should be watched carefully, and increased if it produces no effect. For a child of twelve years, begin with fifteen grains daily, and note the effect of each dose. The drug should be frequently discontinued for a day or so, as it seems to lose its effect.

I merely offer this as the result of observation in one epidemic, for I know that the value of this treatment is acknowledged by some and denied by others.

---

**Diphtheria.**

Dr. H. V. Sweringen, of Fort Wayne, Ind., writes to the *Druggists' Circular* upon the treatment of diphtheria as follows:

The discovery which I think I have made—and it remains for future experience to establish its value—is, that *prompt cinchonism, followed by an alternative tonic, is, if not absolutely a specific, the most proper and successful treatment for diphtheria.*

It may be said, however, that quinine as a remedy in diphtheria is not new; that it is very frequently given in the course of its treatment. This is true; but it has never been given with any very specific object in view, other than its tonic or antiperiodic effect. The remedies considered of the most value in the treatment of this disease are the muriated tincture of iron, chlorate of potassa, carbolic acid, and nitrate of silver,—the former three given both constitutionally and locally, the latter applied locally only. All the text-books which I have consulted seem, in addition to good ventilation, food, and all the necessary hygienic conditions, to rely chiefly upon the above-named remedies,

But it is the condition known as cinchonism which is produced by the admistration of quinine in positive doses, until its peculiar physiological effects are induced to a marked degree, that I contend is the first grand object to be accomplished in the treatment of diphtheria. This statement is based upon the confidence I have in the antiseptic properties of quinine, if properly administered, and the theory I have, that, when the condition of cinchonism is fully established, the septic poison in the circulation is neutralized, and no longer retains its power of exudation upon mucous surfaces; by virtue of this fact the exudation, or false membrane previously formed, loses its vitality or tenacity, and drops off or becomes detached from the throat, or upon whatever mucous surface it may have been attached. This theory is founded on the *fact* that in every case I have thus treated, just as soon as that condition was established, the exudation or false membrane become detached without any local interference whatever.

I observed the fact, also, that the most severe and malignant cases I had, were those in whom it was most difficult to establish the condition of chinchonism. A little boy, 6 years of age—son of C. L. Thomas, Esq., residing at No. 138 Jackson Street, this city—took 64 grains of quinine in forty-eight hours before he complained of ringing in his ears or of deafness; but, when this did take place, the exudation became detached of its own accord, his appetite returned, the swelling of the submaxillary glands began to subside, and in a comparatively short time he made a good recovery. I did not make a single local application in this case. In fact, I have abandoned local treatment, except in those cases where it is absolutely necessary in order to remove excessive obstruction to the air-passages, threatening life by asphyxia, or where it is necessary to correct fetor by the application of disinfectants. I had six other cases under treatment about the same time, all of whom were well marked, and the line of treatment pursued in each was as follows:

(For a child 6 years old:)

℞
Quin. sulph., grs. xxxij.
Acid. tannic, grs. x.
Syr. simp., f. ℥ j.
Tr. ol. menth. pip., gtt. iij.
M. ft. mist.

Sig.—A teaspoonful every three hours until cinchonism is induced.

After which (or it may be administered alternately with the above) the following is given.

℞

Potassii iodidi, grs. xxxij.

Potassii bromidi, ʒ ij.

Syr. simp. } āā f. ℥ j.

Tr. cinch. co. }

M. ft. sol.

Sig.—A teaspoonful every three hours.

Alum or ipecac as emetics are useful when the exudation shows a disposition to extend to the larynx, or when there is much difficulty of breathing from the tumefaction of the fauces, or from the accumulation of the pseudo-membranous deposits.

If the principles involved in the foregoing considerations of the treatment of diphtheria be correct, may we not reasonably conclude that the same, or similar treatment, will prove highly beneficial in cases of childbed fever and erysipelas?

